# Merkel Cells as Putative Regulatory Cells in Skin Disorders: An In Vitro Study

**DOI:** 10.1371/journal.pone.0006528

**Published:** 2009-08-11

**Authors:** Nicholas Boulais, Ulysse Pereira, Nicolas Lebonvallet, Eric Gobin, Germaine Dorange, Nathalie Rougier, Christophe Chesne, Laurent Misery

**Affiliations:** 1 University of Brest, EA4326, Brest, France; 2 University Hospital, Laboratory of Pathology, Brest, France; 3 BIOPREDIC International, Rennes, France; 4 University Hospital, Department of Dermatology, Brest, France; Tufts University, United States of America

## Abstract

Merkel cells (MCs) are involved in mechanoreception, but several lines of evidence suggest that they may also participate in skin disorders through the release of neuropeptides and hormones. In addition, MC hyperplasias have been reported in inflammatory skin diseases. However, neither proliferation nor reactions to the epidermal environment have been demonstrated. We established a culture model enriched in swine MCs to analyze their proliferative capability and to discover MC survival factors and modulators of MC neuroendocrine properties. In culture, MCs reacted to bFGF by extending outgrowths. Conversely, neurotrophins failed to induce cell spreading, suggesting that they do not act as a growth factor for MCs. For the first time, we provide evidence of proliferation in culture through Ki-67 immunoreactivity. We also found that MCs reacted to histamine or activation of the proton gated/osmoreceptor TRPV4 by releasing vasoactive intestinal peptide (VIP). Since VIP is involved in many pathophysiological processes, its release suggests a putative regulatory role for MCs in skin disorders. Moreover, in contrast to mechanotransduction, neuropeptide exocytosis was Ca^2+^-independent, as inhibition of Ca^2+^ channels or culture in the absence of Ca^2+^ failed to decrease the amount of VIP released. We conclude that neuropeptide release and neurotransmitter exocytosis may be two distinct pathways that are differentially regulated.

## Introduction

Merkel cells (MCs) are cutaneous neuroendocrine cells that are mainly found in touch-sensitive areas of the glabrous epidermis and outer root sheaths of hair follicles [1], [2], [3]. MCs are discerned from keratinocytes by their close connections to sensory neurons, the presence of dense-core neurosecretory granules and expression of cytokeratin (CK) 20. Their relationship with nerve terminals and their particular location have led to the hypothesis that MCs function in mechanoreception [4]. Furthermore, convincing evidences have demonstrated their excitability [5], expression of fundamental proteins of the synaptic machinery [6], synaptic-like contact with sensory neurons [7], [8] and involvement of the Ca^2+^-induced Ca^2+^ release (CICR) pathway in the production of Ca^2+^ transients [9], [10], [11].

In addition, MCs produce bioactive amines and peptides such as serotonin, met-enkephalin, chromogranin A, calcitonin gene-related peptide (CGRP) and vasoactive intestinal peptide (VIP), which are held in dense-core granules [12], [13]. Thus, they belong to the neuroendocrine cell family. It is widely accepted that neuroendocrine cells participate in tissue homeostasis, cell growth and regeneration. The presence of non-innervated MCs in hair follicles [14] and in oral mucosa [15] fosters the idea that MCs assume such functions. Finally, MCs are thought to stimulate keratinocyte proliferation, to regulate skin homeostasis and the hair cycle and to play a trophic role toward sensory neurons [16], [17]. Unfortunately, the role of MCs in the cutaneous environment remains largely unexplored.

MCs appear to be quite scarce in the epidermis, which has hampered their study in vitro. Analyses performed in total epidermal cell cultures revealed that they are difficult to maintain for more than two weeks [18], [19]. MCs were not labeled by BrdU [20], they did not incorporate ^3^H-thymidine [21] and they were not immunoreactive to Ki-67 [22], [23], an antigen that is associated with proliferating cells. Thus, in contrast to neighboring keratinocytes, they apparently do not renew. However, MC hyperplasias have been reported in some inflammatory skin diseases [24], such as actinic keratosis [25], neurodermatitis [26] and fibrous papules [27], which suggests that they proliferate under certain conditions and that they have a role in skin disorders.

In the skin, VIP is produced by MCs and some peptidergic nerve terminals [28], [29]. VIP was shown to enhance the proliferation of keratinocytes [30], to induce mast cell degranulation [31] and to enhance IL-1α, IL-6, IL-8, RANTES and TNF-α expression by keratinocytes [32] [33]. Conversely, VIP exhibits immuno-modulatory activity by decreasing the production of pro-inflammatory cytokines [34], [35]. Since VIP expression is upregulated during psoriasis [36], atopic dermatitis [37] (with conflicting results) and skin disorders like aquadynia [38], better knowledge of the mediators leading to VIP release by MCs is of great interest for the understanding of these diseases.

To gain further insights into the neuroendocrine functions of MCs, we developed cultures enriched in MCs from swine snouts. This cell type is enriched in the swine snout. Different growth factors and molecules were compared. We show here that, in the presence of serum and a low proportion of keratinocytes, MCs were able to proliferate. In culture, MCs reacted to histamine and were able to sense tissue acidification and cell swelling through the transient receptor potential vanilloid (TRPV) type 4 receptor. These conditions induced the release of VIP. Conversely, acetylcholine (Ach) inhibited neuropeptide exocytosis. Moreover, in contrast to the dependence of their mechanotransduction properties on Ca^2+^ signaling, neuropeptide release involved a (Ca^2+^)-independent secretory pathway that was inhibited by Ca^2+^.

## Results

### Enrichment for MCs

**Figure 1:**
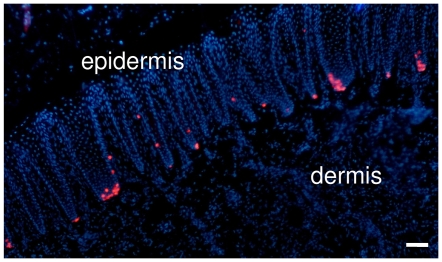
MC are densely represented in the swine snout. MCs labeled by CK20 immunostaining (in red) appear abundant in the basal layer of the epidermis of swine snout. Conversely, less than 50 MCs/mm^2^ were found in human glabrous skin. Nuclei were stained in blue with DAPI. (Scale bar, 50 µm).

MCs are rare neuroendocrine cells of the epidermis. Due to their rarity, a great challenge to their study is the harvest of a sufficient number of cells to perform experiments. A significant advance was achieved by the purification of GFP-positive MCs from transgenic mice [6], [39], but this method remains restrictive for most laboratories. Here, we used a positive magnetic cell sorting strategy to enrich for MCs. The swine snout was preferred to human biopsies for its higher proportion of MCs as demonstrated by CK20 immunostaining (Figure 1).

**Figure 2:**
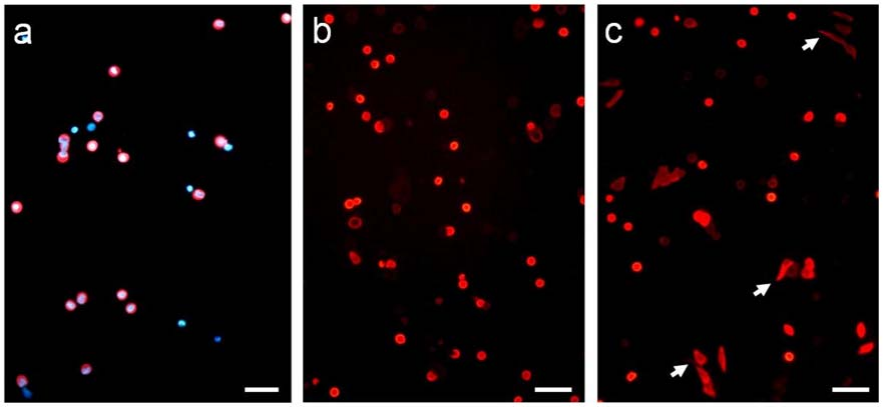
Immunofluorescence on selected Merkel cells in culture. (a) After enrichment, an average of 62% (±3.5) MCs was obtained over 10 experiments as demonstrated by immunostaining using anti-CK20 antibodies and DAPI as a counterstain. In order to define a suitable culture medium for MCs, cells were stimulated by various factors and the morphology of MCs was analyzed by CK20 immunofluorescence. Using DMEM/F12 as a basal medium, neither EGF (20 ng/mL), RA (0.5 µM) or Dexa (1 µM) supported the spreading of MCs. Similarly, neither NGF (100 ng/mL), BDNF (25 ng/mL), NT-3 (25 ng/mL) or the three factors together (b) stimulated the growth of cytoplasmic extensions. (c) Conversely, MCs reacted to bFGF (20 ng/mL), as they extended cytoplasmic outgrowths (arrows), suggesting that this factor acts as a growth factor for MCs. (Scale bar in all pictures, 50 µm).

**Figure 3:**
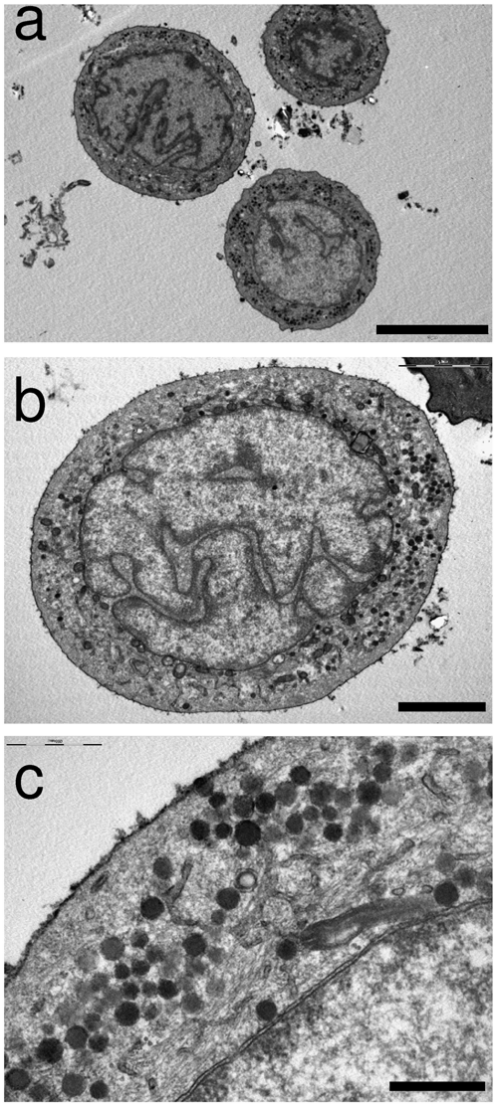
Electron microscopy analysis confirmed the identity of Merkel cells. Ultrastructural analyses were carried out on cells from the enriched MC fraction. (a, b) Up to half of the cells presented features characteristic of MCs: a polylobulated nucleus with numerous typical dense-core granules in a clear cytoplasm. (c) The thin membrane distinguishable around the darkest cytoplasmic granules is consistent with neuroendocrine cells. (Scale bar in a, 5 µm; in b, 2 µm; in c, 500 nm).

An average of 45.7 (±13)×10^6^ dissociated epidermal cells was obtained per swine snout. We targeted CD56 antigen for the purpose of cell sorting. It is one of the only extracellular markers described for human and swine MCs [40], [41], although all MCs do not express it [42]. We routinely obtained ∼5.9×10^5^ cells (1.54±0.15% of the total dissociated cells) in the CD56-positive fraction, of which MCs accounted for an average of 62% (±3.5) as shown in over 10 experiments employing CK20 immunofluorescence and DAPI counterstaining (Figure 2a). In contrast, CK20-positive cells were rarely observed in the unlabeled fraction. To confirm this result, collected cells were post-fixed in glutaraldehyde buffer and observed by electron microscopy. The ultrastructural analysis confirmed the predominance of small cells with large, polylobulated nuclei and clear cytoplasm containing dense-core granules of an average diameter of 100 nm (Figure 3). This ultrastructure is characteristic of MCs [1], [43], which demonstrates the ability of this method to enrich for MCs.

### Merkel cells in culture

Culture of MCs has been reported to be very difficult. Under previously reported culture conditions, cells underwent apoptosis within two weeks and no proliferation was observed [7], [20]. However, MC hyperplasia has been reported; therefore, MCs can proliferate in response to specific factors. We exposed cultured MCs to different media, growth factors and mediators. The medium was changed every two days. MCs were analyzed after three days by immunofluorescence. The keratinocyte growth medium (K-SFM) did not allow MCs to develop cytoplasmic extensions. Furthermore, this culture method led to a rapid decrease in the proportion of MCs due to keratinocyte proliferation. Therefore, DMEM/F12 was used as a basal medium. The addition of EGF (20 ng/mL) also failed to affect MCs, as they remained round in shape. This result was consistent with their neural crest origin [44]. However, none of NGF (100 ng/mL), NT-3 (25 ng/mL), BDNF (25 ng/mL) or all three together modified MC behavior (Figure 2b). Similarly, MCs were not affected by RA (0.5 µM).

MC hyperplasia occurs in some inflammatory diseases, but culture with IL-6 or TNF-α did not lead to MC proliferation [7]. Hence, we cultured MCs with glucocorticoid (Dexa, 1 µM) to evoke growth arrest and differentiation with the aim of observing cell spreading. However, Dexa did not induce extension of cytoplasmic outgrowths. By contrast, once stimulated by bFGF (20 ng/mL), MCs clearly developed cytoplasmic processes and became easily distinguishable from keratinocytes (Figure 2c). These morphological changes reflected the viability of the cells and indicated that bFGF is a likely survival factor for MCs.

### Merkel cells can proliferate in vitro

**Figure 4:**
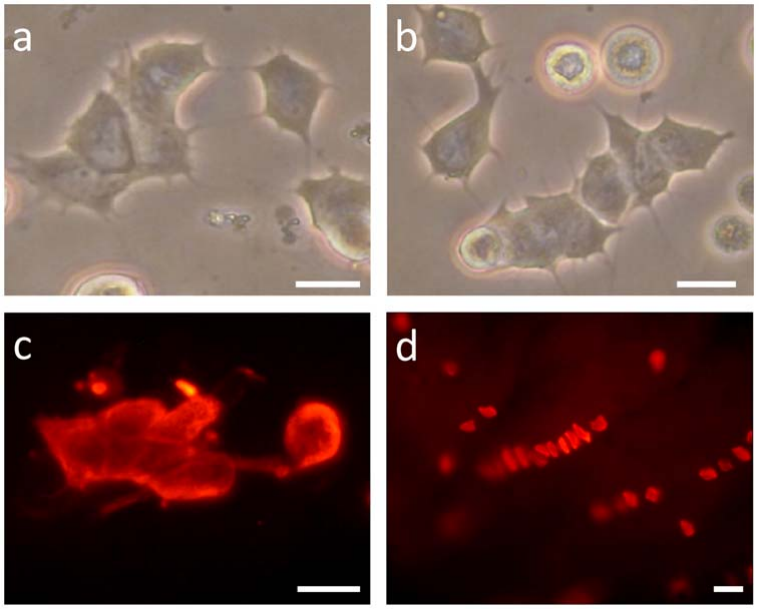
MCs developed cytoplasmic processes when cultured in the presence of serum. Strikingly, in these culture conditions, stacking of more than five MCs was observed (a, b) and confirmed by CK20 immunofluorescence (c). Interestingly, this stacking is similar to configurations observed in mechanosensitive areas in the touch pads of rats, as demonstrated by CK20 immunostaining in dissociated epidermal layers (d). The meaning of these alignments to the role of MCs remains to be defined. (Scale bars, 10 µm).

Finally, we cultured MCs in DMEM/F12 serum-supplemented medium (7% FCS and 7% HS). After three days, most of the cells assumed a dendritic shape more readily than with bFGF. In this condition, MCs established numerous connections between one another, and several stacks of dendritic MCs were observed (Figure 4a, b). This particular configuration was confirmed by immunofluorescence against CK20 (Figure 4c). Strikingly, this particular arrangement evoked the configuration that MCs physiologically take in areas sensitive to touch, like in the footpads of rodents, as we found by immunohistochemistry of dissociated epidermal layers (Figure 4d).

**Figure 5:**
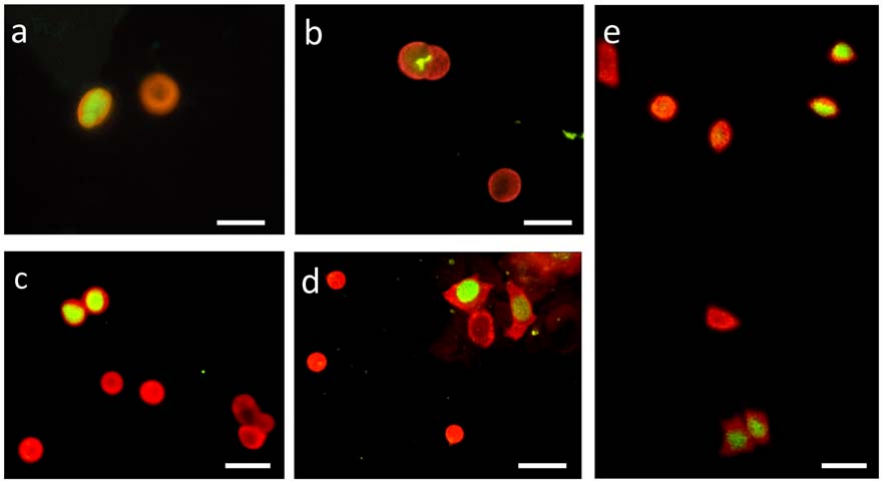
Merkel cells proliferated in culture. Double immunofluorescence assay using antibodies against CK20 (red) and Ki-67 antigen (green) revealed the ability of MCs to proliferate in vitro. Dividing CK20-positive cells were clearly visible, and all phases of the cell cycle were observed: prophase (a), metaphase (b), anaphase (c, e) and telophase (d, e). (Scale bars, 20 µm).

Therefore, we addressed the question of how such arrangements develop. Specific cell migrations were contested and MCs are thought to be terminally differentiated so they may not proliferate [7], [22], [23]. In the present study, we tested the immunoreactivity of cultured MCs to the proliferation marker Ki-67. Unexpectedly, many cells were positive for both CK20 and Ki-67.All phases of mitosis were visible: prophase (Figure 5a), metaphase (Figure 5b), anaphase (Figure 5c, e) and telophase (Figure 5d, e). MCs were followed for over four weeks in culture, but proliferating CK20-positive cells were detected only during the first two weeks after enrichment. These results demonstrate for the first time that MCs can undergo mitosis in vitro. We supposed that keratinocyte factors disable this capability, which would explain why previous reports failed to amplify MCs.

### MCs produce VIP in culture

**Figure 6:**
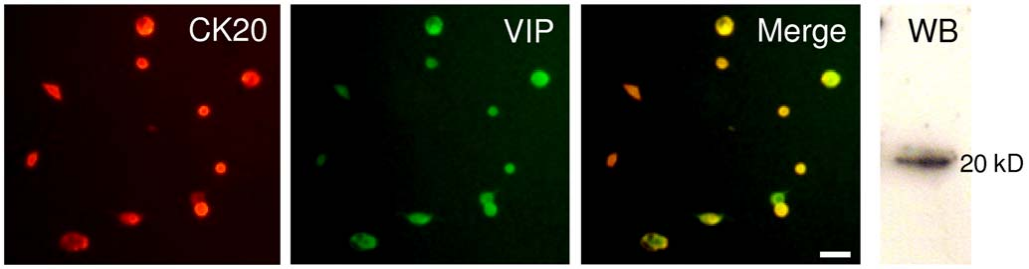
Merkel cells produced VIP in culture. In our culture conditions, MCs produced the neuropeptide VIP as demonstrated by double immunofluorescence using antibodies against CK20 (red) and VIP (green). The production of VIP was confirmed by western blot analysis (WB). (Scale bar, 50 µm).

MCs of most mammalian species produce VIP [45]. Conversely, keratinocytes do not express it under physiological conditions [38]. In our culture conditions, MCs were immunoreactive to VIP as revealed by double immunofluorescence (Figure 6). The merge picture demonstrated that nearly all CK20-positive MCs also produced VIP, and that cells other than MCs were not found to express VIP. In addition, the synthesis of VIP was detected by western blot (WB) analysis of whole cell homogenates produced from cultures enriched in MCs (Figure 6, WB). In contrast, calcitonin gene-related peptide (CGRP) and substance P were produced by MCs and keratinocytes. Neuropeptide Y (NPY) was hardly detectable in our cultures (data not shown).

### Ca^2+^ signaling in neuropeptide release from MCs

MCs were shown to express the histamine H3 receptor [46] and TRPV4 [47], and Ca^2+^ signaling was demonstrated to be an essential component in mechanoreception [9]. We analyzed how these factors modulate neuropeptide exocytosis from MCs by monitoring the amount of VIP released into the culture medium by ELISA. Cells were stimulated in DMEM/F12, which contains 1 mM Ca^2+^, as a control condition. Supernatant was harvested and analyzed after 5 min. The basal level was defined as 100%.

**Figure 7:**
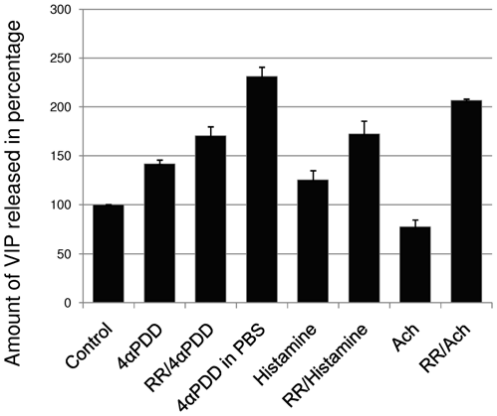
Percentage of VIP released by Merkel cells. The amount of VIP released in the culture supernatant, assessed by ELISA and expressed as a percentage of the control conditions (DMEM/F12 containing 1 mM Ca^2+^). Exposure to the TRPV4 agonist 4αPDD (1 µM) or histamine (100 µM) stimulated VIP release from MCs. This release was not inhibited by pre-incubation with the Ca^2+^ channel inhibitor RR (1 µM), indicating a Ca^2+^-independent pathway. The marked increase of VIP release obtained following stimulation by 4αPDD in a Ca^2+^-free buffer confirmed these results. In addition, exposure to the neurotransmitter Ach (10 µM) significantly inhibited the amount of VIP released in the presence of Ca^2+^. This inhibition was suppressed by pre-incubation with RR (1 µM), suggesting the involvement of Ca^2+^ channels (n = 4, difference significant at P≤0.025.).

Activation of TRPV4 by its specific agonist, 4α-phorbol-12,13-didecanoate (4αPDD) (1 µM), enhanced VIP release by 42%, while stimulation by histamine (100 µM) led to an increase of 25% (Figure 7, difference was significant at P≤0.025, n = 4). Thus, MCs react to histamine and are able to perceive tissue acidification and cell swelling through TRPV4.

To study the involvement of Ca^2+^ signaling in neuropeptide release, a 5-min pre-incubation was performed with the Ca^2+^ channel inhibitor ruthenium red (RR) (1 µM). Unexpectedly, this treatment increased the VIP concentration in the supernatant following histamine (+72%) as well as to 4αPDD (+70%) stimulation. To confirm this result, we stimulated MCs by treatment with 4αPDD in Ca^2+^-free PBS. In this case, a dramatic enhancement of VIP release was measured (+131%). These results strongly suggest that dense-core granule exocytosis occurs via Ca^2+^-independent signaling. Moreover, activation of Ca^2+^ signaling seems to interfere with VIP release.

In some neurons and neuroendocrine cells in which both dense-core granules and synaptic vesicles co-exist, an intracellular pathway other than Ca^2+^ signaling can modulate exocytosis [48]. This pathway exists in neurons that co-produce Ach as a neurotransmitter and neuropeptides such as VIP. In such a configuration, Ach has been reported to inhibit VIP release through the muscarinic Ach receptor [49], [50]. To find out whether MCs respond like these cells, we stimulated them with Ach. Addition of Ach (10 µM) to the culture medium reduced the release of VIP by 23%; however, pre-inhibition of Ca^2+^ channels with RR (1 µM) before Ach stimulation enhanced VIP release by 107% (Figure 7, n = 4). These results are consistent with signaling via the muscarinic Ach receptor.

**Figure 8:**
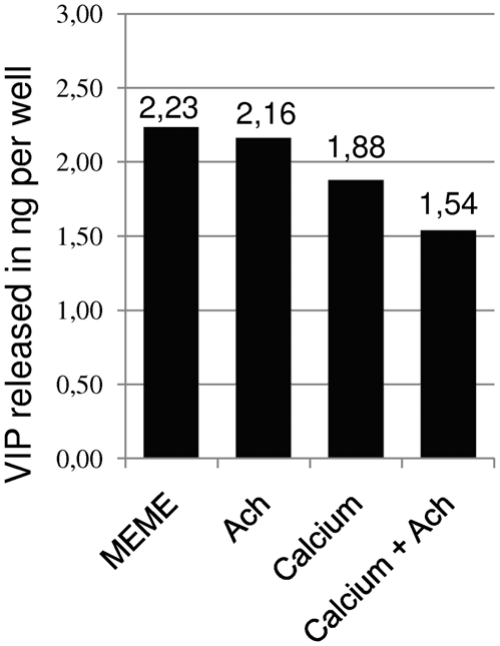
Ca^2+^ and Ach decreased the release of VIP. Effect of Ach and Ca^2+^ on VIP release expressed in ng/well. MCs were cultured in Ca^2+^-free MEME and then exposed to Ach (100 µM), Ca^2+^ (1 mM), or both. In the absence of Ca^2+^, the addition of Ach failed to significantly decrease VIP exocytosis (P>0.1). A lower amount of VIP release was observed in the presence of Ca^2+^ (1 mM). Finally, the inhibitory effect of Ach on VIP release required extracellular Ca^2+^ (P≤0.025). These results are consistent with inhibition via Ca^2+^ channels, probably via muscarinic M2 or M4 Ach receptors.

To explore this phenomenon further, we stimulated MCs in Ca^2+^-free medium (MEME). In this condition, Ach failed to inhibit VIP release significantly (Figure 8, P>0.1, representative results from two experiments). The addition of Ca^2+^ (1 mM) to the culture medium was sufficient to reduce this amount to a lower level (−16%), which corroborates the previous results (Figure 7 and 8). Stimulation by Ach in the presence of Ca^2+^ markedly inhibited VIP exocytosis (−31%), thereby confirming the involvement of Ca^2+^ signaling in the inhibition of neuropeptide release.

## Discussion

Consistent with their neural crest origin [44], MCs were not found to renew like keratinocytes [20], [21], [22]. Thus, they are believed to have a long life span, similar to neurons. MCs act in mechanoreception, but their exact role remains to be identified. They are thought to participate in skin homeostasis through the release of bioactive molecules, but few reports have focused on the neuroendocrine properties of MCs. This lack of knowledge is mainly due to the lack of an experimental model. We developed a method for generating cultures enriched in porcine MCs. In the process of this work, we identified in vitro survival factors and demonstrated that MCs are able to proliferate in culture. Subsequently, we found that the MCs reacted to histamine and activation of TRPV4. These stimulations led to VIP release in a Ca^2+^-independent manner. Conversely, Ach inhibited VIP release, probably through the activation of Ca^2+^ channels.

Previous attempts to culture MCs were performed in total epidermal cell cultures in which keratinocytes were predominant. In these cultures, MCs were difficult to maintain for more than two weeks, they did not undergo mitosis and the low number of MCs restricted the opportunities for analysis [7], [18], [51]. To overcome these obstacles, E. A. Lumpkin and her team used transgenic mice from which GFP-positive MCs were efficiently selected by fluorescence-activated cell sorting (FACS) [6]. Here, we used a magnetic cell sorting system to select MCs from swine snout, as this tissue has one of the highest densities of MCs among mammalian tissues (Figure 1). Compared to FACS selection, we achieved a lower ratio of MCs in our cultures (60–65% versus 85–95%), but a higher number of MCs per animal. In addition, this technique has been successfully used to enrich human MCs, but with a yield proportional to the lower initial MC density in this tissue.

Through morphological analysis by fluorescence microscopy, we observed MC spreading in the presence of bFGF. The extension of outgrowths reflected the activity of a growth factor. This finding suggests involvement of the bFGF pathway in MC hyperplasia and possibly in Merkel cell carcinoma (MCC). By contrast, keratinocyte growth factors did not stimulate the development of MC extensions. This finding could be consistent with their neural crest origin; however, all of the tested neurotrophins failed to support the development of cytoplasmic extensions (Figure 2b). This result is surprising given that the nerve dependency of MCs in vivo has been reported several times. First, mice lacking receptors for NGF, BDNF and NT-3 (TrkA, TrkB and TrkC respectively) have a lower number of MCs than control mice [52], [53]. Also, NT-3 appears not to be essential for the development of MCs, but its absence leads to perinatal apoptosis of MCs [54]. Finally, overexpression of NT-3 and BDNF increased the number of MCs [55], [56]. In fact, all these mutations also affect the cutaneous nerve density, which correlates with MC number. Our in vitro results seem to demonstrate that neurotrophins alone are not sufficient to maintain MCs in culture. Hence, other paracrine factors from sensory nerve endings probably assume this function.

When cultured in serum-supplemented medium, alignment of MCs was observed (Figure 4a, b). To the best of our knowledge, this configuration has not been reported before. Interestingly, in vitro staking of MCs can be compared to the in vivo staking of MCs detected in rodent touch pads (Figure 4d), and perhaps they imply the same events. The proliferative ability of MCs has been a matter of debate for decades. Based on Ki-67 immunoreactivity, we provide direct evidence that MCs can undergo mitosis in vitro (Figure 5), although most studies have concluded that they are terminally differentiated. We hypothesize that the predominance of epidermal factors regulates this capability, as suggested by our results (see above). Thus, an environment rich in MC growth factors, as occurs in MC hyperplasia, could support the proliferation of MCs. In our model, MCs underwent mitosis during the first two weeks, before overgrowth by keratinocytes. Further analysis of the signaling pathway that initiates primary MC mitosis would be of interest in the study of MCC. The recently identified MC polyomavirus [57] integrated in most MCCs probably interferes with this pathway.

We demonstrated here that MCs react to histamine and to TRPV4 stimulation by releasing VIP (Figure 7). Thus, in the skin, MCs would be able to respond to mast cell degranulation, cell swelling and tissue acidification. Since VIP released in the epidermal environment is known to have immuno-modulatory functions and effects on keratinocytes, our findings support a regulatory role of MCs in skin diseases. VIP differentially regulates immune functions according to the tissue and the disease, and its role in skin disorders has yet to be detailed. To our knowledge, these data are the first to support a participatory role of MCs in skin pathophysiology.

The level of released VIP was lower for cells cultured in DMEM:F12 (Ca^2+^, 1 mM) compared to those cultured in PBS without Ca^2+^ (Figure 7). In order to avoid interactions with components of the culture medium that are not present in PBS, we compared the amounts of VIP released in MEME (a medium without Ca^2+^) with or without the addition of Ca^2+^ (Figure 8), and we found similar results. These data contrast with the Ca^2+^-dependence of the synaptic vesicle exocytosis underlying mechanotransduction. Thus, two distinct secretory pathways are indicated. First, a Ca^2+^-dependent signaling pathway activates the CICR pathway, which leads to neurotransmitter release in the mechanotransduction process [9], [10], [11]. Second, a Ca^2+^-independent pathway is involved in dense-core granule exocytosis. Our data support the hypothesis that activation of Ca^2+^-dependent signaling inhibits the Ca^2+^-independent pathway involved in neuropeptide release, since inhibition of the first one increased the level of activation of the second one.

In neurons and neuroendocrine cells where two secretory pathway co-exist, exocytosis could be regulated or modulated by intracellular signals such as cAMP or diacylglycerol [49]. This pathway remains to be clarified for neuropeptide release from MCs. Furthermore, Consistent with previous studies that have described interference between Ach exocytosis as a neurotransmitter and VIP release [50], we demonstrated in MCs that Ach inhibits VIP release in a Ca^2+^-dependent manner, probably through M2 or M4 muscarinic Ach autoreceptors [49]. Based on these results, we propose Ach as a putative neurotransmitter for sensory nerve endings, since met-enkephalin and glutamate did not satisfy this function [58], [59].

In conclusion, this work describes a new way to culture of MCs. In our experimental model, we provide evidence that MCs can undergo mitosis. Through the release of VIP following histamine stimulation and activation of TRPV4, MCs probably act in skin pathophysiology, but their exact role remains to be defined. The proliferation of MCs in particular conditions could amplify these effects. In addition, the mechanotransduction properties of MCs appear to be independent of neuropeptide exocytosis, as the latter does not require Ca^2+^ signaling. Finally, we characterized the role of Ach in neurosecretory release.

## Materials and Methods

### Cell cultures enriched in Merkel cells

Swine snouts were retrieved from the local slaughterhouse. Only young swine aged 2–6 days were used. The epidermal layer was separated from the dermis by enzymatic digestion with dispase (15 U/mL, 37°C; Gibco, Paisley, UK). Epidermal cells were dissociated by digestion with 0.05% trypsin-EDTA (Lonza, Walkersville, MD). Retrieved cells were passed through a 30-µm mesh nylon filter, washed twice in chilled phosphate-buffered saline (PBS) supplemented with 2 mM EDTA and 0.5% fetal bovine serum (FBS; PAA Laboratories, Pasching, Austria), and then immunolabeled using microbead-conjugated antibody against CD56. Cells were isolated using the magnetic cell sorting system Mini-MACS (Miltenyi Biotec, Bergisch Gladbach, Germany). Viable trypan-blue excluding cells were counted by use of a hemacytometer. Data were expressed as means±standard deviation. The media used in cell cultures were Dulbecco's Modified Eagle Medium/Ham's F-12 (DMEM/F12) (Lonza), keratinocyte-serum free medium (K-SFM) (Gibco), and Minimum Essential Medium Eagle (MEME) (Sigma-Aldrich, St Louis, MO). Fetal calf serum (FCS) and horse serum (HS) were purchased from PAA Laboratories. Normocin™ (100 µg/mL) (Invivogen, San Diego, CA) was added as an antibiotic. Epidermal growth factor (EGF, 20 ng/mL), basic fibroblast growth factor (bFGF, 20 ng/mL), nerve growth factor (NGF, 100 ng/mL), neurotrophin-3 (NT-3, 25 ng/mL), brain-derived neurotrophic factor (BDNF, 25 ng/mL), retinoic acid (RA, 0.5 µM), and dexamethasone (Dexa, 1 µM) were obtained from Sigma-Aldrich.

### Immunofluorescence

Cells were seeded on sterile Biocoat CultureSlides coated with laminin and poly-d-lysine (Becton–Dickinson, Heidelberg, Germany) at 5×10^4^ cells per slide. Cells were fixed in 4% paraformaldehyde, permeabilized with 0.5% Triton X-100, saturated in 5% normal goat serum (NGS) in 0.1% Tween-20 in PBS (PBS-T) and hybridized with primary antibodies overnight at 4°C in PBS-T, 1% NGS. After two washes, cells were hybridized for two hours with secondary antibodies. Nuclei were stained for 10 minutes with 4′,6-diamidino-2-phenylindole dissolved to 100 ng/mL in PBS. The fluorescence analyses were performed using a BX41 Olympus upright microscope. Pictures were taken with an Olympus C-5060 digital camera. The following antibodies were used: mouse monoclonal anti-CK20 (1∶100; Progen GmbH, Heidelberg, Germany), rabbit polyclonal anti-Ki-67 (1∶200; Abcam, Cambridge, UK), goat polyclonal anti-VIP (1∶50; Santa Cruz Biotechnology, Santa Cruz, CA), mouse monoclonal anti-human CD56 (1∶10; Miltenyi Biotech), goat polyclonal anti-mouse IgG specific for Fab and conjugated to TRITC (1∶300; Sigma-Aldrich), peroxidase-conjugated donkey polyclonal anti-goat antibody (1∶2,000; Santa Cruz), and goat polyclonal anti-rabbit IgG specific for F(ab') and conjugated to FITC (1∶500; Jackson ImmunoResearch, West Grove, PA).

### Immunohistochemistry

Pieces of skin biopsies were embedded in OCT, cryopreserved in isopentane chilled on liquid nitrogen and sections with a thickness of 10 µm were cut. Slides were saturated with 5% NGS in PBS with 0.05% Triton X-100 for 15 minutes and subsequently hybridized with primary antibodies. Slides were rinsed twice and hybridized with appropriate secondary antibodies for two hours at room temperature. The antibodies used were those described above.

### Electron microscopy

MC-enriched suspensions were fixed in 2.5% glutaraldehyde for 30 minutes, rinsed in 0.1 M cacodylate buffer, and post-fixed for one hour with 1% OsO_4_. Cells were dehydrated in graded concentrations of alcohol, and finally by propylene oxide. Then, cells were embedded in epoxy resin for one day at 60°C. Finally, ultrathin sections were cut and observed with a Jeol, Jem-1010 Electron Microscope.

### Analysis of neuropeptide release

VIP is produced by MCs of most mammalian species [28]. Its release was studied by western blot (WB) and ELISA. MCs were seeded at 5×10^4^ cells per well in 24-well tissue culture plates for three days in growth medium. Cells were washed twice and then exposed to histamine (100 µM), Ach (10 µM), 4 αPDD (1 µM) or Ca^2+^ (1 mM) in DMEM-F12, PBS or MEME with or without Ca^2+^. In some cases, cells were pre-exposed for 5 minutes to the Ca^2+^ channel antagonist ruthenium red (1 µM). All chemicals were purchased from Sigma-Aldrich. After a 5-minute incubation, culture supernatants were collected and centrifuged to remove detached cells. For WB analysis, supernatants were diluted in Laemmli buffer (100 mM Tris/HCl, 4% SDS, 15% glycerol, 15% 2-mercaptoethanol, 20 µg/mL bromophenol blue). Proteins were separated by 12% SDS-PAGE and hybridized to nitrocellulose membranes (PALL). The membranes were proved with the indicated antibodies. Proteins were detected by chemiluminescence using the Western Blotting Luminol Reagent (Santa Cruz). For ELISA, 50 µL of supernatant were assessed in triplicate in 96-well ELISA microplates (Greiner Bio One). The wells were saturated with 3% BSA in PBS-T over-night in order to allow for complete binding. Hybridizations with primary and secondary antibodies were carried out for two hours in PBS-T 0.3% BSA. The amount of neuropeptide was assessed using the SureBlue Reserve TMB Microwell Peroxidase substrate (KPL). To measure the amount of VIP release, the blocking peptide VIP (Santa Cruz) was used as a standard. A nonparametric Mann-Whitney test was used for statistical analyses.

## References

[1] Moll I, Roessler M, Brandner JM, Eispert AC, Houdek P (2005). Human Merkel cells–aspects of cell biology, distribution and functions.. Eur J Cell Biol.

[2] Halata Z, Grim M, Bauman KI (2003). Friedrich Sigmund Merkel and his “Merkel cell”, morphology, development, and physiology: review and new results.. Anat Rec A Discov Mol Cell Evol Biol.

[3] Boulais N, Misery L (2007). Merkel cells.. J Am Acad Dermatol.

[4] Ogawa H (1996). The Merkel cell as a possible mechanoreceptor cell.. Prog Neurobiol.

[5] Yamashita Y, Akaike N, Wakamori M, Ikeda I, Ogawa H (1992). Voltage-dependent currents in isolated single Merkel cells of rats.. J Physiol.

[6] Haeberle H, Fujiwara M, Chuang J, Medina MM, Panditrao MV (2004). Molecular profiling reveals synaptic release machinery in Merkel cells.. Proc Natl Acad Sci U S A.

[7] Shimohira-Yamasaki M, Toda S, Narisawa Y, Sugihara H (2006). Merkel cell-nerve cell interaction undergoes formation of a synapse-like structure in a primary culture.. Cell Struct Funct.

[8] Chateau Y, Dorange G, Clement JF, Pennec JP, Gobin E (2007). In vitro reconstruction of neuro-epidermal connections.. J Invest Dermatol.

[9] Senok SS, Baumann KI (1997). Functional evidence for calcium-induced calcium release in isolated rat vibrissal Merkel cell mechanoreceptors.. J Physiol.

[10] Piskorowski R, Haeberle H, Panditrao MV, Lumpkin EA (2008). Voltage-activated ion channels and Ca(2+)-induced Ca (2+) release shape Ca (2+) signaling in Merkel cells.. Pflugers Arch.

[11] Haeberle H, Bryan LA, Vadakkan TJ, Dickinson ME, Lumpkin EA (2008). Swelling-activated Ca2+ channels trigger Ca2+ signals in Merkel cells.. PLoS ONE.

[12] Hartschuh W, Weihe E, Yanaihara N (1989). Immunohistochemical analysis of chromogranin A and multiple peptides in the mammalian Merkel cell: further evidence for its paraneuronal function?. Arch Histol Cytol.

[13] Gaudillere A, Misery L (1994). Merkel cell.. Ann Dermatol Venereol.

[14] Narisawa Y, Hashimoto K, Kohda H (1994). Merkel cells of the terminal hair follicle of the adult human scalp.. J Invest Dermatol.

[15] Tachibana T, Yamamoto H, Takahashi N, Kamegai T, Shibanai S (1997). Polymorphism of Merkel cells in the rodent palatine mucosa: immunohistochemical and ultrastructural studies.. Arch Histol Cytol.

[16] Tachibana T (1995). The Merkel cell: recent findings and unresolved problems.. Arch Histol Cytol.

[17] Moll I, Paus R, Moll R (1996). Merkel cells in mouse skin: intermediate filament pattern, localization, and hair cycle-dependent density.. J Invest Dermatol.

[18] Fradette J, Larouche D, Fugere C, Guignard R, Beauparlant A (2003). Normal human Merkel cells are present in epidermal cell populations isolated and cultured from glabrous and hairy skin sites.. J Invest Dermatol.

[19] Kim DK, Holbrook KA (2001). The nerve-dependency of Merkel cell proliferation in cultured human fetal glabrous skin.. Yonsei Med J.

[20] Vaigot P, Pisani A, Darmon YM, Ortonne JP (1987). The majority of epidermal Merkel cells are non-proliferative: a quantitative immunofluorescence analysis.. Acta Derm Venereol.

[21] Merot Y, Saurat JH (1988). Proliferation of Merkel cells in the skin.. Acta Derm Venereol.

[22] Moll I, Zieger W, Schmelz M (1996). Proliferative Merkel cells were not detected in human skin.. Arch Dermatol Res.

[23] Tachibana T, Fujiwara N, Nawa T (2000). Postnatal differentiation of Merkel cells in the rat palatine mucosa, with special reference to the timing of peripheral nerve development and the potency of cell mitosis.. Anat Embryol (Berl).

[24] Tachibana T, Kamegai T, Takahashi N, Nawa T (1998). Evidence for polymorphism of Merkel cells in the adult human oral mucosa.. Arch Histol Cytol.

[25] Merot Y, Mooy A (1989). Merkel cell hyperplasia in hypertrophic varieties of actinic keratoses.. Dermatologica.

[26] Regazzini R, De Filippi C (1995). Merkel cell hyperplasia in circumscribed neurodermatitis: a quantitative study.. Eur J Dermatol.

[27] Kanitakis J, Bourchany D, Faure M, Claudy A (1998). Merkel Cells in Hyperplastic and Neoplastic Lesions of the Skin.. Dermatology.

[28] Hartschuh W, Reinecke M, Weihe E, Yanaihara N (1984). VIP-immunoreactivity in the skin of various mammals: immunohistochemical, radioimmunological and experimental evidence for a dual localization in cutaneous nerves and merkel cells.. Peptides.

[29] Boulais N, Misery L (2008). The epidermis: a sensory tissue.. Eur J Dermatol.

[30] Granoth R, Fridkin M, Gozes I (2000). VIP and the potent analog, stearyl-Nle(17)-VIP, induce proliferation of keratinocytes.. FEBS Lett.

[31] Kulka M, Sheen CH, Tancowny BP, Grammer LC, Schleimer RP (2008). Neuropeptides activate human mast cell degranulation and chemokine production.. Immunology.

[32] Dallos A, Kiss M, Polyanka H, Dobozy A, Kemeny L (2006). Effects of the neuropeptides substance P, calcitonin gene-related peptide, vasoactive intestinal polypeptide and galanin on the production of nerve growth factor and inflammatory cytokines in cultured human keratinocytes.. Neuropeptides.

[33] Kakurai M, Fujita N, Murata S, Furukawa Y, Demitsu T (2001). Vasoactive intestinal peptide regulates its receptor expression and functions of human keratinocytes via type I vasoactive intestinal peptide receptors.. J Invest Dermatol.

[34] Fernandez-Martin A, Gonzalez-Rey E, Chorny A, Martin J, Pozo D (2006). VIP prevents experimental multiple sclerosis by downregulating both inflammatory and autoimmune components of the disease.. Ann N Y Acad Sci.

[35] Delgado M, Munoz-Elias EJ, Gomariz RP, Ganea D (1999). Vasoactive intestinal peptide and pituitary adenylate cyclase-activating polypeptide prevent inducible nitric oxide synthase transcription in macrophages by inhibiting NF-kappa B and IFN regulatory factor 1 activation.. J Immunol.

[36] Reich A, Szepietowski JC (2008). Vasoactive peptides in the pathogenesis of psoriasis.. G Ital Dermatol Venereol.

[37] Fantini F, Pincelli C, Romualdi P, Donatini A, Giannetti A (1992). Substance P levels are decreased in lesional skin of atopic dermatitis.. Exp Dermatol.

[38] Misery L, Meyronet D, Pichon M, Brutin JL, Pestre P (2003). [Aquadynia: a role for VIP?].. Ann Dermatol Venereol.

[39] Lumpkin EA, Collisson T, Parab P, Omer-Abdalla A, Haeberle H (2003). Math1-driven GFP expression in the developing nervous system of transgenic mice.. Gene Expr Patterns.

[40] Gallego R, Garcia-Caballero T, Fraga M, Beiras A, Forteza J (1995). Neural cell adhesion molecule immunoreactivity in Merkel cells and Merkel cell tumours.. Virchows Arch.

[41] McNiff JM, Cowper SE, Lazova R, Subtil A, Glusac EJ (2005). CD56 staining in Merkel cell carcinoma and natural killer-cell lymphoma: magic bullet, diagnostic pitfall, or both?. J Cutan Pathol.

[42] Eispert AC, Fuchs F, Brandner JM, Houdek P, Wladykowski E (2009). Evidence for distinct populations of human Merkel cells.. Histochem Cell Biol.

[43] Cheng Chew SB, Leung PY (1994). Ultrastructural study of the Merkel cell and its expression of met-enkephalin immunoreactivity during fetal and postnatal development in mice.. J Anat.

[44] Szeder V, Grim M, Halata Z, Sieber-Blum M (2003). Neural crest origin of mammalian Merkel cells.. Dev Biol.

[45] Hartschuh W, Weihe E, Yanaihara N, Reinecke M (1983). Immunohistochemical localization of vasoactive intestinal polypeptide (VIP) in Merkel cells of various mammals: evidence for a neuromodulator function of the Merkel cell.. J Invest Dermatol.

[46] Cannon KE, Chazot PL, Hann V, Shenton F, Hough LB (2007). Immunohistochemical localization of histamine H(3) receptors in rodent skin, dorsal root ganglia, superior cervical ganglia, and spinal cord: Potential antinociceptive targets.. Pain.

[47] Liedtke W, Choe Y, Marti-Renom MA, Bell AM, Denis CS (2000). Vanilloid receptor-related osmotically activated channel (VR-OAC), a candidate vertebrate osmoreceptor.. Cell.

[48] Brosius DC, Hackett JT, Tuttle JB (1992). Ca(2+)-independent and Ca(2+)-dependent stimulation of quantal neurosecretion in avian ciliary ganglion neurons.. J Neurophysiol.

[49] Seino S, Shibasaki T (2005). PKA-dependent and PKA-independent pathways for cAMP-regulated exocytosis.. Physiol Rev.

[50] Bartfai T, Iverfeldt K, Fisone G, Serfozo P (1988). Regulation of the release of coexisting neurotransmitters.. Annu Rev Pharmacol Toxicol.

[51] Vos P, Stark F, Pittman RN (1991). Merkel cells in vitro: production of nerve growth factor and selective interactions with sensory neurons.. Dev Biol.

[52] Ichikawa H, Matsuo S, Silos-Santiago I, Jacquin MF, Sugimoto T (2001). Developmental dependency of Merkel endings on trks in the palate.. Brain Res Mol Brain Res.

[53] Fundin BT, Silos-Santiago I, Ernfors P, Fagan AM, Aldskogius H (1997). Differential dependency of cutaneous mechanoreceptors on neurotrophins, trk receptors, and P75 LNGFR.. Dev Biol.

[54] Szeder V, Grim M, Kucera J, Sieber-Blum M (2003). Neurotrophin-3 signaling in mammalian Merkel cell development.. Dev Dyn.

[55] Krimm RF, Davis BM, Woodbury CJ, Albers KM (2004). NT3 expressed in skin causes enhancement of SA1 sensory neurons that leads to postnatal enhancement of Merkel cells.. J Comp Neurol.

[56] Botchkarev VA, Kief S, Paus R, Moll I (1999). Overexpression of brain-derived neurotrophic factor increases Merkel cell number in murine skin.. J Invest Dermatol.

[57] Feng H, Shuda M, Chang Y, Moore PS (2008). Clonal integration of a polyomavirus in human Merkel cell carcinoma.. Science.

[58] Gottschaldt KM, Vahle-Hinz C (1982). Evidence against transmitter function of met-enkephalin and chemosynaptic impulse generation in “Merkel cell” mechanoreceptors.. Exp Brain Res.

[59] Cahusac PM, Senok SS (2006). Metabotropic glutamate receptor antagonists selectively enhance responses of slowly adapting type I mechanoreceptors.. Synapse.

